# Genetic code expansion and enzymatic modifications as accessible methods for studying site‐specific post‐translational modifications of alpha‐synuclein and tau

**DOI:** 10.1002/pro.70302

**Published:** 2025-09-13

**Authors:** Ibrahim G. Saleh, Marie Shimogawa, Jennifer Ramirez, Bernard Abakah, Yarra Venkatesh, Honey Priya James, Ming‐Hao Li, Sarah A. Louie, Marshall G. Lougee, Wai‐Kit Chia, Christopher Brue, Richard B. Cooley, Ryan A. Mehl, Tobias Baumgart, Robert H. Mach, David Eliezer, Elizabeth Rhoades, E. James Petersson

**Affiliations:** ^1^ Department of Chemistry, School of Arts and Sciences University of Pennsylvania Philadelphia Pennsylvania USA; ^2^ Graduate Group in Biochemistry, Biophysics, and Chemical Biology, Perelman School of Medicine University of Pennsylvania Philadelphia Pennsylvania USA; ^3^ Department of Biochemistry Weill Cornell Medicine New York New York USA; ^4^ Department of Biochemistry and Biophysics Oregon State University Corvallis Oregon USA; ^5^ Department of Radiology Perelman School of Medicine, University of Pennsylvania Philadelphia Pennsylvania USA; ^6^ Department of Biochemistry and Biophysics, Perelman School of Medicine University of Pennsylvania Philadelphia Pennsylvania USA

**Keywords:** alpha‐synuclein, genetic code expansion, post‐translational modification, tau

## Abstract

Alpha‐synuclein (αS) and tau play important roles in the pathology of Parkinson's disease and Alzheimer's disease, respectively, as well as numerous other neurodegenerative diseases. Both proteins are classified as intrinsically disordered proteins (IDPs), as they have no stable structure that underlies their function in healthy tissue, and both proteins are prone to aggregation in disease states. There is substantial interest in understanding the roles that post‐translational modifications (PTMs) play in regulating the structural dynamics and function of αS and tau monomers, as well as their propensity to aggregate. While there have been many valuable insights into site‐specific effects of PTMs garnered through chemical synthesis and semi‐synthesis, these techniques are often outside of the expertise of biochemistry and biophysics laboratories wishing to study αS and tau. Therefore, we have assembled a primer on genetic code expansion and enzymatic modification approaches to installing PTMs into αS and tau site‐specifically, including isotopic labeling for NMR and fluorescent labeling for biophysics and microscopy experiments. These methods should be enabling for those wishing to study authentic PTMs in αS or tau as well as the broader field of IDPs and aggregating proteins.

## 1 INTRODUCTION

**FIGURE 1:**
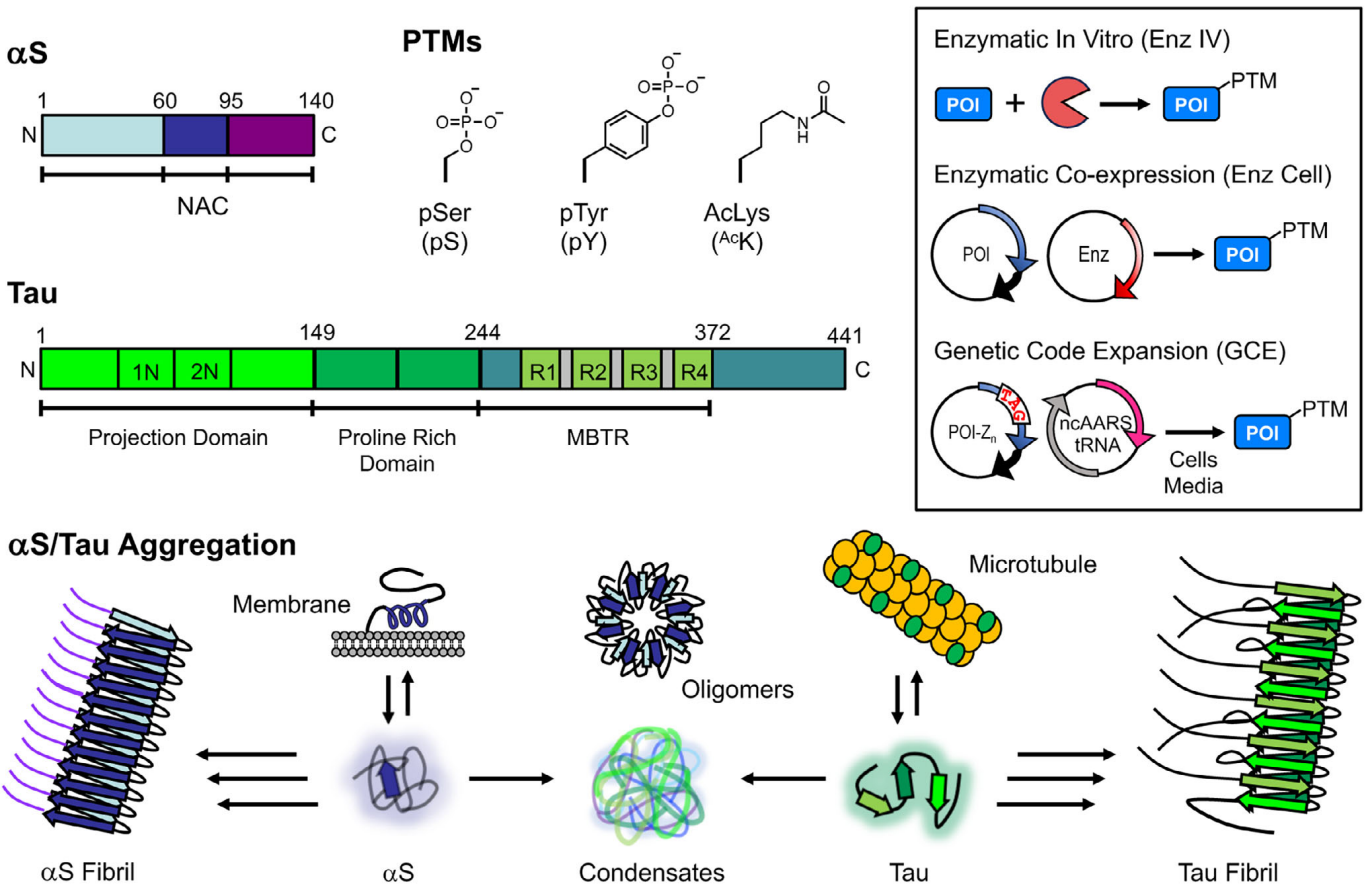
Alpha‐synuclein (αS), Tau, post‐translational modifications (PTMs), and modification methods. Top left: αS and tau structures shown schematically with domains highlighted. PTM structures and abbreviations. Top right (inset): PTM installation methods for protein of interest (POI). Bottom: αS and tau functional states and aggregation pathways.

Alpha‐synuclein (αS) and tau are classified as intrinsically disordered proteins (IDPs) that play crucial roles in cellular functions and are central to the pathology of numerous neurodegenerative diseases, particularly through their aggregation (Figure 1). In healthy contexts, αS exists primarily in the presynaptic terminals of neurons, and its native roles are thought to be in vesicle trafficking and regulating neurotransmission (Burré et al., 2010; Cabin et al., 2002). In pathological contexts, its fibrillar aggregates are found in post‐mortem brain tissue of patients with diseases collectively referred to as synucleinopathies, including Parkinson's disease (PD) and multiple system atrophy (MSA). Tau is a microtubule‐associated protein found primarily in the axons of neurons and is thought to be involved in microtubule stabilization and axonal transport (Kempf et al., 1996). Tauopathies, of which Alzheimer's disease (AD) is the most common, feature neuronal tau fibrils (Lee et al., 2001). There is also significant interest in non‐fibrillar aggregates of αS and tau, including soluble oligomers and condensates (Figure 1). Critical to the functionality and dysregulation of IDPs are post‐translational modifications (PTMs), particularly phosphorylation of Ser, Thr, or Tyr and acetylation of Lys (Figure 1), which can influence interactions with other biomolecules and the formation of pathological assemblies. Understanding the precise impact of site‐specific PTMs on IDPs such as αS and tau is crucial for elucidating their roles in health and disease.

A large number of PTMs have been observed on αS and shown to affect its functional interactions with vesicles and pathological aggregation (Hassanzadeh et al., 2024; Pancoe et al., 2022; Ramirez et al., 2023). Of these modifications, phosphorylation is one of the most prominent. Ser129 phosphorylation (pS_129_) is a hallmark of pathology, found in 90% of aggregated αS but only 4% of its soluble form, and is thought to disrupt dopamine uptake and inhibit αS binding to synaptic vesicles (Hara et al., 2013). While antibodies directed to pS_129_ are the standard in the field for observing PD and MSA pathology in immunohistochemistry, there are still questions as to its role in affecting αS function and aggregation. Tyr39 phosphorylation (pY_39_) has been associated with conflicting reports showing both increased neurodegeneration and slowed fibrillization kinetics in different studies (Brahmachari et al., 2016; Dikiy et al., 2016; Mahul‐Mellier et al., 2014). Inhibitors targeting the kinase responsible for pY_39_, c‐Abl, have undergone human trials as PD therapeutics, motivating a need for a deeper understanding of pY_39_ effects on αS (Berton et al., 2023). Ser87 phosphorylation (pS_87_) has been shown to prevent αS fibril formation by increasing its flexibility as a monomer (Paleologou et al., 2010). Cryo‐electron microscopy (cryo‐EM) structures of αS fibrils composed of αS‐pY_39_ or αS‐pS_87_ have been solved, showing dramatic conformational changes relative to structures of unmodified fibrils (Hu et al., 2024; Zhao et al., 2020). However, these structures were determined with 100% modified protein, while studies of phosphorylation levels in patient tissue have shown that they are only present on 10%–25% of αS (Zhang et al., 2023). Thus, in spite of these studies clearly showing the impact of PTMs on αS function and pathology, there remain many unanswered questions needing further investigation.

PTMs have also been shown to play roles in the protein levels and aggregation of tau (Park et al., 2018). Moreover, various PTMs have been detected in tau from healthy brains, suggesting their involvement in tau's normal microtubule‐associated functions (Haj‐Yahya & Lashuel, 2018). Indeed, about 35% of tau's amino acid residues are susceptible to modification (Alquezar et al., 2020). One particular PTM of interest for tau is lysine acetylation, where recent reports have pointed to sex‐specific impacts of Lys PTMs on tau. Despite women having higher tau levels and an increased risk of AD compared to men (Collaborators GBDDF, 2022), the exact cause of this heightened susceptibility is not yet understood. It has been hypothesized that tau levels are regulated by an X‐chromosome‐linked deubiquitinase that controls ubiquitin‐mediated degradation through modification of Lys 274 and 281 (Yan et al., 2022). Tau acetylation should also play a regulatory role, since ubiquitination at these residues occurs in competition with lysine acetylation (^Ac^K). Additionally, ^Ac^K_274_, ^Ac^K_280_, and ^Ac^K_281_ are associated with impaired microtubule binding and increased tau aggregation (Cohen et al., 2011; Gorsky et al., 2016; Haj‐Yahya & Lashuel, 2018; Min et al., 2010). Mass spectrometry (MS) identified Lys280 as a major site of tau acetylation in diseased tissue, suggesting its role in pathological tau transformation (Cohen et al., 2011). Collectively, these studies prompt investigations of these three ^Ac^K sites to determine how much of the impact of acetylation comes from direct functional modulation versus its ability to block ubiquitination.

Traditionally, biochemists and biophysicists have studied PTMs through a strategy of mutating the target sites to other naturally occurring amino acids that mimic the desired PTM. Commonly used “phosphomimetics” include glutamate or aspartate; however, they lack the dianionic nature of the phosphate group (Wojciechowski et al., 2003) and are less sterically bulky than tyrosine, which can result in different properties compared to phosphotyrosine (Pan et al., 2020). To investigate acetylation, lysine is often mutated to glutamine, but this involves a significant reduction in sidechain length. These mimics, while easy to introduce, often do not reproduce the effects of the authentic PTM, particularly in IDPs like αS and tau, where single point mutations can produce dramatic changes in folding and dynamics (Pancoe et al., 2022).

The study of authentic, site‐specific PTMs in proteins has largely been approached through various methods including site‐specific mutagenesis, chemical or semi‐synthesis, and enzymatic reactions, each with inherent limitations. While mutagenesis allows for facile introduction of mutation‐based mimics of PTMs, these alterations do not always recapitulate the chemical and structural nuances of naturally occurring PTMs (Pan et al., 2020). On the other hand, the use of peptide ligation techniques, although precise, often requires sophisticated synthetic expertise and can be resource and time intensive (Moon et al., 2021). Enzymatic methods are limited by the availability of specific, efficient enzymes, but have proven successful in certain instances where site specificity and yield satisfy the experimental needs (Johnson et al., 2010; Pan et al., 2020; Ramirez et al., 2024). It must be noted that enzymatic modification often requires additional mutations to eliminate off‐target introduction of PTMs, such as Ser‐to‐Ala or Tyr‐to‐Phe mutations to prevent phosphorylation, and these mutations can introduce artifacts (Balasuriya et al., 2018). We highlight cases involving αS where enzymatic methods have effectively introduced PTMs, either through in vitro enzymatic treatment (Enz IV) of the purified protein or through co‐expression of the enzyme in cells (Enz Cell) by co‐transformation of a plasmid for the protein of interest (POI) and a plasmid for the enzyme (Figure 1, inset).

For broader applicability and ease of use, genetic code expansion (GCE) is a robust alternative (Costello et al., 2024). GCE involves the targeted incorporation of noncanonical amino acids (ncAAs) into proteins, typically by mutation to introduce a TAG (amber) stop codon at the site of interest. Delivery of the ncAA is achieved through an orthogonal transfer RNA (tRNA_CUA_), which recognizes the TAG codon, and an engineered orthogonal aminoacyl‐tRNA synthetase (RS) designed or evolved for the specific ncAA of interest (Dumas et al., 2015). GCE incorporation of the PTM is then performed by co‐transformation of a plasmid for the POI with a TAG codon at the PTM site and a plasmid for the ncAARS and tRNA_CUA_, sometimes in combination with specialized cells or media (Figure 1, inset). Cooley and Mehl have developed an efficient phosphoserine GCE system (pSer‐3.1G) using a robust release factor 1 deficient *Escherichia coli* strain to minimize truncation issues that can arise from reassignment of the TAG stop codon (Allen et al., 2024; Zhu et al., 2019). For ^Ac^K incorporation, we use an RS system developed by Liu and coworkers using phage‐assisted continuous evolution (Bryson et al., 2017; Esvelt et al., 2011), chAcK3RS with IPYE mutations, based on the *Methanosarcina barkeri* pyrolysyl RS (Neumann et al., 2008). Although neither system has been previously used with αS and tau, it was relatively easy to adapt them for use with these proteins, highlighting the versatility of the GCE approach.

**FIGURE 2:**
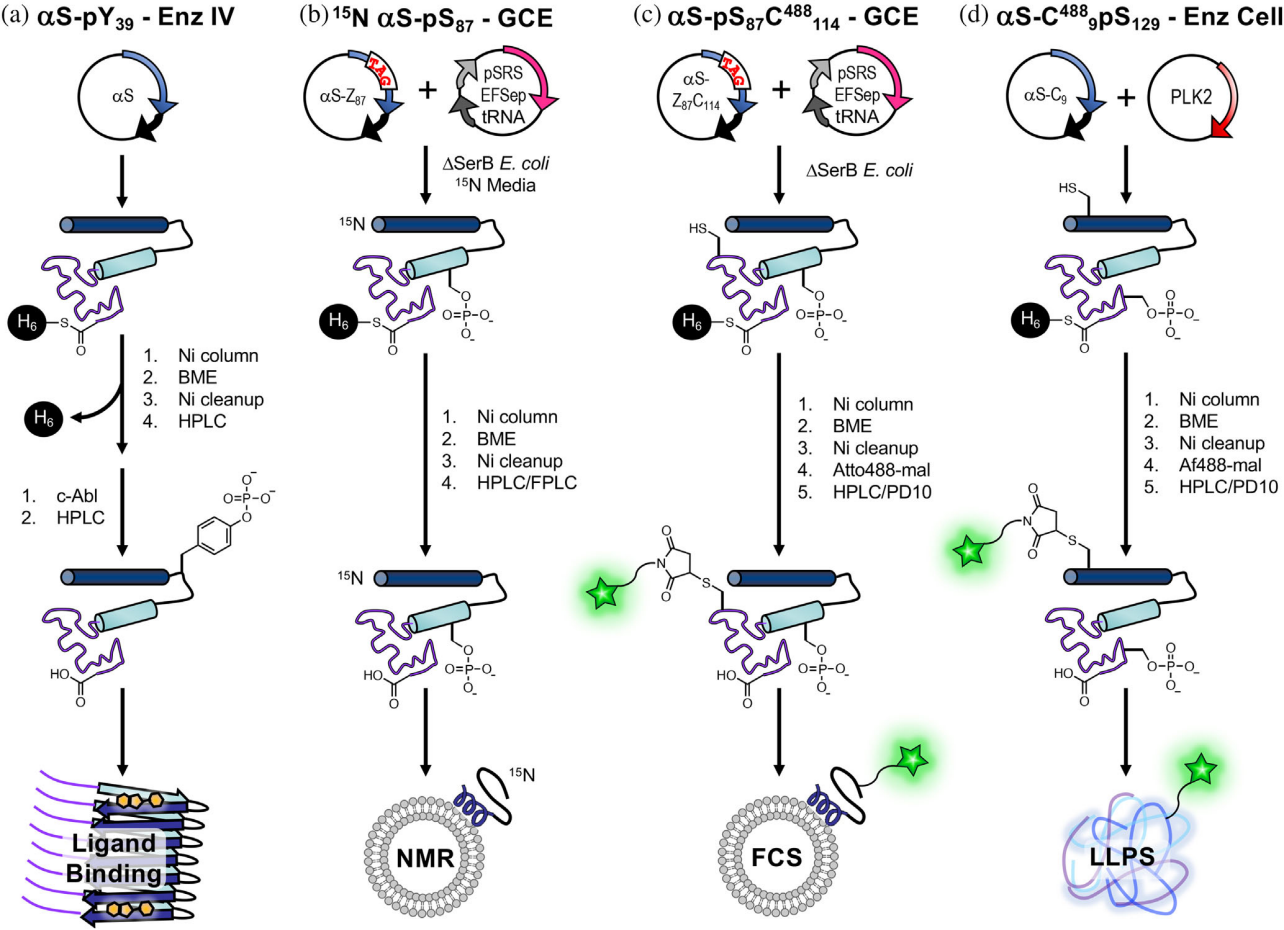
Introduction of post‐translational modifications (PTMs) and labels to alpha‐synuclein (αS) for diverse applications. Workflows are shown for (a) in vitro enzymatic treatment (Enz IV) for ligand binding beta‐mercaptoethanol (BME), (b) genetic code expansion (GCE) including ^15^N labeling for NMR, (c) GCE including fluorescent labeling for fluorescence correlation spectroscopy (FCS), and (d) enzyme in cells (Enz Cell) and fluorescent labeling for liquid/liquid phase separation (LLPS) microscopy. Intein six His (H_6_) tags are used for initial purification followed by more intensive high performance liquid chromatography/fast protein liquid chromatography (HPLC/FPLC) purification or simpler PD10 column purification for dye removal.

Herein, we showcase the use of either enzymatic modifications or GCE to study four PTM targets (Figures 2 and 4): (i) pS_87_ in αS (GCE); (ii) pY_39_ in αS (Enz IV); (iii) pS_129_ in αS (GCE and Enz Cell); and (iv) ^Ac^K_274_, ^Ac^K_280_, and ^Ac^K_281_ in tau (GCE). We then characterize how these PTMs affect factors critical in αS and tau native or pathological contexts using exemplary assays: αS‐vesicle binding through fluorescence correlation spectroscopy (FCS) and nuclear magnetic resonance (NMR), αS fibril conformation through radioligand binding, tau‐mediated tubulin polymerization, and phase transition through fluorescence microscopy of mixed αS/tau condensates formed by liquid/liquid phase separation (LLPS). By integrating successful enzymatic modifications with GCE, we aim to provide guidance for biochemists and biophysicists who wish to achieve a nuanced understanding of authentic PTM impacts on IDPs. First, we will describe the preparation and characterization of authentically labeled protein, then demonstrate various experimental uses of these proteins to test the effects of the PTMs.

## 2 PRODUCTION OF SITE‐SPECIFICALLY MODIFIED PROTEINS THROUGH GCE AND ENZYMATIC METHODS

### 2.1 Production of phosphorylated αS by GCE

Using methods similar to those that we have previously described for recombinantly expressing αS with ncAAs in *E. coli*, one can produce αS with phosphorylation of serine 87 (αS‐pS_87_) as shown in Figure 2. We use two plasmids, one encoding the aaRS for phosphoserine and its cognate tRNA as well as EFSep, an EF‐Tu mutant that better accommodates delivery of pSer‐aminoacylated tRNA, and the other encoding the POI with a TAG stop codon at the site of ncAA incorporation. Often, our POI bears a C‐terminal traceless intein‐His_6_ tag, which was cleaved off with thiols during purification to provide scarless protein products and allows for isolation of full‐length products from species truncated at the TAG codon (Batjargal et al., 2015).

**FIGURE 3:**
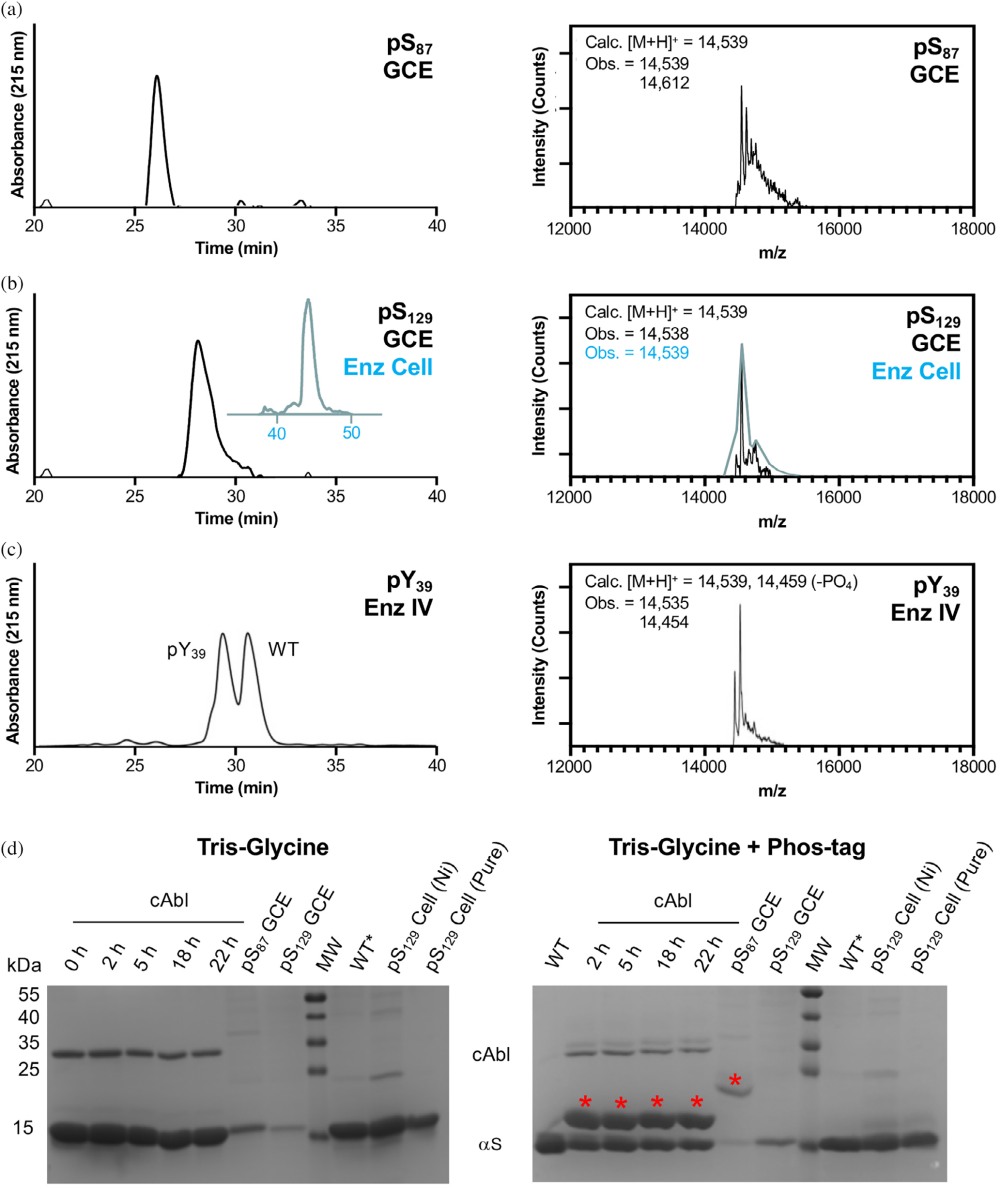
Characterization of phosphorylated alpha‐synuclein (αS). High performance liquid chromatography (HPLC) and matrix‐assisted laser desorption ionization mass spectrometry for (a) Ser87 phosphorylation (pS_87_) by genetic code expansion (GCE), (b) Ser129 phosphorylation (pS_129_) by GCE and enzyme in cells (Enz Cell), and (c) Tyr39 phosphorylation (pY_39_) by Enz IV. αS‐pS_129_ from cellular expression (teal) was analyzed using a different high performance liquid chromatography (HPLC) gradient than αS‐pS_129_ from GCE (black). (d) Phos‐tag gel analysis of αS constructs after treatment of purified wild‐type (WT) with c‐Abl for varying amounts of time, after purification from GCE expression, or after purification from enzymatic co‐expression in cells. WT* indicates N‐terminally acetylated αS, produced by enzymatic expression as previously described. αS‐pS_129_ produced by enzymatic co‐expression was tested after Ni column elution, and also after additional HPLC purification. Red asterisks indicate bands for phosphorylated αS on the Phos‐tag gel.

For pSer GCE, *E. coli* cells with a ΔserB phosphatase genomic knockout (BL21 ΔserB or B95 ΔserB cells developed by Zhu et al. 2019, the latter lacks release factor 1) were used so that pSer is accumulated intracellularly and readily available for GCE, since pSer cannot be exogenously added due to its low cell permeability. Following affinity purification, high performance liquid chromatography (HPLC) was used to separate the product from impurities, which were characterized by matrix‐assisted laser desorption ionization (MALDI) mass spectrometry (MS) and found to include de‐phosphorylation (−80 Da), Gln misincorporation, and an unknown +72 Da species (Figures 3 and S3). We found BL21 ΔserB cells to be higher yielding than B95 ΔserB cells, with a two‐fold higher level of protein expression and slightly lower fraction of impurities. As a method to determine the homogeneity of phosphorylated samples, we performed Phos‐tag gel electrophoresis, where the phosphate‐binding ligand usually slows the mobility of phosphorylated proteins (Kinoshita et al., 2022). We observed a mobility shift of αS‐pS_87_, where the extent of the shift seems to depend slightly on buffer components (Figure S1, see marked bands in “Ni cleanup” and “HPLC purified”). We observed nearly quantitative isolation of phosphorylated product from the unphosphorylated species by HPLC (Figures 3 and S2, HPLC, Figure S3: MALDI‐MS of HPLC‐purified product).

We expressed αS‐pS_129_ using the pSer‐3.1G system as well, and similarly observed a mixture of products, with the predominant species being the desired phosphorylated protein (Figure 3). However, unlike αS‐pS_87_, the αS‐pS_129_ species could not easily be separated by HPLC, and yields (10.2 mg/L) were limited by the need to select fractions based on MALDI‐MS analysis to obtain pure phosphorylated protein. Interestingly, we did not observe significant amounts of the +72 Da species for incorporation at Ser129. In spite of MALDI‐MS data indicating the presence of pSer, we observed no shift on the Phos‐tag gel for αS‐pS_129_ (Figure 3), a finding corroborated by the production of αS‐pS_129_ through co‐expression with a kinase (see below). This is likely due to the acidic nature of the C‐terminal region of αS.

To demonstrate the power of the GCE method over native chemical ligation based (NCL) incorporation, we additionally prepared a fluorescently labeled αS‐pS_87_ construct for FCS and an isotopically labeled variant of αS‐pS_87_ for NMR. For FCS, fluorescently labeled αS variants with a Cys mutation at site 114 were prepared by site‐directed mutagenesis and reacted with Atto488‐maleimide overnight at room temperature, resulting in a labeled, phosphorylated construct (αS‐pS_87_C^488^_114_) in 0.5 mg/L yield. This illustrates the value of the GCE approach, as we had previously synthesized αS‐pS_87_C^488^_114_ by NCL, but obtained yields of <100 μg, which were too low for full characterization of the vesicle binding curve (Galesic et al., 2023). For NMR, we prepared ^15^N isotopically labeled αS‐pS_87_, a construct that was prohibitively expensive to produce by NCL due to the high cost of isotopically labeled amino acids for solid‐phase peptide synthesis. The ^15^N αS‐pS_87_ expression followed strategies introduced by Vesely et al. (2022)—it was necessary to start the culture with rich media and then switch to minimal media so that Ser auxotroph *E. coli* ΔserB cells were kept alive while minimizing ^14^N incorporation. The proteins were expressed at lower temperatures, but for longer, to minimize the activity of endogenous phosphatases. HPLC was again used to purify phosphorylated αS (0.3 mg/L yield) from de‐phosphorylated side products, wherein we observed that a larger fraction of αS was de‐phosphorylated in the ^15^N isotopic labeling protocol (Figure S2: HPLC, Figure S3: MALDI‐MS of HPLC‐purified product).

### 2.2 Production of phosphorylated αS by enzymatic modification

For comparison to GCE, production of αS‐pS_129_ was also performed as in previous reports from the Rhoades laboratory (Ramirez et al., 2024), by co‐expressing αS with Ser/Thr kinase PLK2 in *E. coli*. This is fortunately made possible by utilizing the enzymatic activity of PLK2 to phosphorylate Ser129 selectively and quantitatively without posing toxicity to *E. coli*. This method gives a very high yield (6.9 mg/L yield) and therefore for this phosphorylation site, this was our method of choice over the GCE approach. Again, we observed no mobility shift of this phosphorylated product on Phos‐tag gel when tested either immediately after Ni column elution (to minimize any potential phosphate hydrolysis or elimination) or after further purification (Figure 3). To generate fluorescently labeled αS‐pS_129_ for microscopy studies, we expressed αS‐C_9_pS_129_ using the PLK co‐expression method (5.9 mg/L yield), purified the protein using a Ni column, and reacted it with Alexafluor 488‐maleimide, followed by HPLC purification to generate αS‐C^488^_9_pS_129_ (2.6 mg/L yield), which was characterized by MALDI‐MS (Figure S6).

Production of αS‐pY_39_ was performed using an in vitro, chemoenzymatic approach with a recombinantly expressed catalytic domain of the tyrosine kinase c‐Abl, similar to our published approach. It has been noted that in prokaryotes like *E. coli*, c‐Abl needs to be co‐expressed with the phosphatase YopH to counteract and suppress its toxicity, and this makes it impossible to co‐express the kinase with αS and get Tyr39 phosphorylated at appreciable levels in *E. coli* cells (Pan et al., 2021). In previous reports, we used a semi‐synthetic approach to ensure that αS was phosphorylated only at Tyr39. Here, in order to avoid the need for protein ligation, we performed enzymatic modification on full‐length αS. We chose to do this because we were able to achieve ~50% conversion and site selectivity of c‐Abl for pY_39_. Upon incubation of αS with c‐Abl, we observed a mobility shift for ~50% of αS on Phos‐tag gel, with confirmatory analysis by HPLC and MALDI‐MS (Figure 3). Following Glu‐C digestion and MALDI‐MS analysis, we observed only the αS_36‐46_ peptide as a phosphorylated peptide, where MS/MS analysis showed that Tyr39 was phosphorylated (Figure S5). As one can see in the HPLC trace in Figure 3, αS‐pY_39_ can be separated from unmodified αS if one wishes to analyze 100% phosphorylated material (note: separation can be performed by analytical HPLC, but obtaining pure αS‐pY_39_ may not be possible at the semi‐prep scale). In our case, we were interested in more physiologically relevant levels of phosphorylation (10%–25% at Tyr39, as determined from analyses of patient samples) (Zhang et al., 2023), so we simply mixed the 50% modified protein with unmodified αS prior to aggregation to form fibrils.

### 2.3 Production of acetylated tau by GCE

**FIGURE 4:**
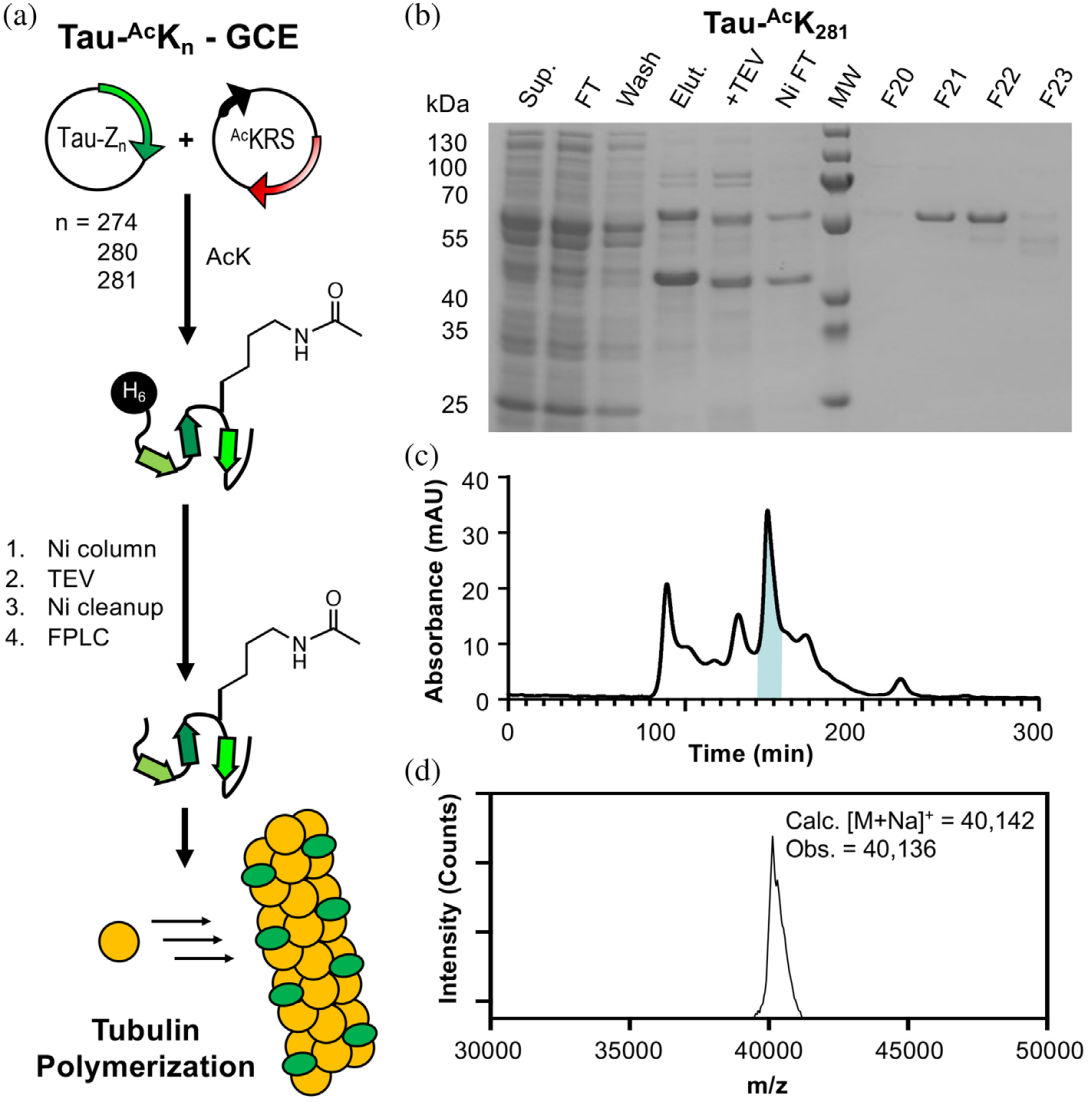
Expression and analysis of tau‐^Ac^K_281_ construct. (a) Expression of acetylated tau constructs and use in tubulin polymerization assays. (b) Gel analysis of expression and purification. (c) fast protein liquid chromatography purification. Highlighted fractions correspond to F20 to F23 shown on gel above. (d) Matrix‐assisted laser desorption ionization mass spectrometry analysis of F21 and F22 confirming the identity of tau‐^Ac^K_281_. GCE, genetic code expansion; TEV, tobacco etch virus.

To produce acetylated tau constructs, plasmids encoding the 0N4R tau variant with a TAG mutation at Lys274, Lys280, or Lys281 were generated by site‐directed mutagenesis. Tau 0N4R was selected as the isoform of interest due to its abundance in the adult human brain. Each tau TAG mutant plasmid was co‐transformed into BL21 *E. coli* cells with the plasmid encoding the chAcK3RS/tRNA_CUA_ pair and expressed in media with ^Ac^K and 50 mM nicotinamide (to suppress deacetylases). The tau plasmid used in these experiments has an N‐terminal His tag with a tobacco etch virus (TEV) cleavage site. After Ni column purification, the His tag was cleaved with TEV and the tau ^Ac^K constructs were purified by fast protein liquid chromatography (FPLC) using a size exclusion column to obtain tau‐^Ac^K_274_, tau‐^Ac^K_280_, and tau‐^Ac^K_281_ in 1.1, 1.2, and 1.2 mg/L yields, respectively. Employing two‐round FPLC purification may allow for the collection of purer fractions. Acetylation was confirmed via MALDI‐MS and electospray ionization MS to be ≥95% for all three constructs (Figures 4 and S7). Additionally, protease digests were performed for all three acetylated tau variants as well as wild‐type (WT) tau, and LCMS‐MS data confirmed the incorporation of all ^Ac^K site‐specifically (Figure S13).

With these purified, PTM‐modified αS and tau constructs, we then measured αS‐pS_87_ vesicle binding through FCS and NMR, studied αS‐pY_39_ fibril conformation through radioligand binding, probed tau acetylation impacts on tubulin polymerization, and imaged the effects of pS_129_ on αS/tau condensate formation.

## 3 ASSESSING EFFECTS OF SITE‐SPECIFIC PTMs

### 3.1 Effects of pY_39_ on radioligand binding to αS fibrils

Radioligands, such as positron emission tomography (PET) tracers, are powerful tools for imaging αS aggregates in tissues and in vivo that can provide insights into misfolding and aggregation in PD and MSA. The Petersson and Mach laboratories have developed PET probes to bind selectively and with high affinity to specific sites on αS fibrils in order to visualize disease‐associated aggregates (Ferrie et al., 2020; Hsieh et al., 2018). Binding sites were computationally and experimentally mapped based on the original solid state NMR structure (Protein Data Bank, PDB ID: 2n0a) published by Rienstra and coworkers, which is similar to many unmodified αS fibril structures published to date, including those solved by cryo‐EM (Pancoe et al., 2022). Building on this, one could take radioligand PET probes with known binding sites in unmodified αS fibrils (Tg‐190b for “Site 2” and BF‐2846 for “Site 9”) and investigate their binding to post‐translationally modified αS fibrils, then use this information to better understand the conformational differences introduced by specific modifications. Since PTMs can significantly alter αS fibril structure and behavior, radioligand binding provides a functional readout that can hint at how these modifications influence fibril conformation. αS‐pY_39_ was of particular interest, not only because Tyr39 is within Site 2, the target of Tg‐190b binding, but also because Zhao et al. showed through protein synthesis and cryo‐EM that the structure of 100% αS‐pY_39_ fibrils (PDB IDs: 6l1t and 6l1u) is dramatically different from unmodified, WT fibrils (Zhao et al., 2020). However, since physiological αS‐pY_39_ levels range from 10% to 25% (Zhang et al., 2023), we were interested in whether these lower levels of PTM modification induced a conformational change that could be detected by radioligand binding.

**FIGURE 5:**
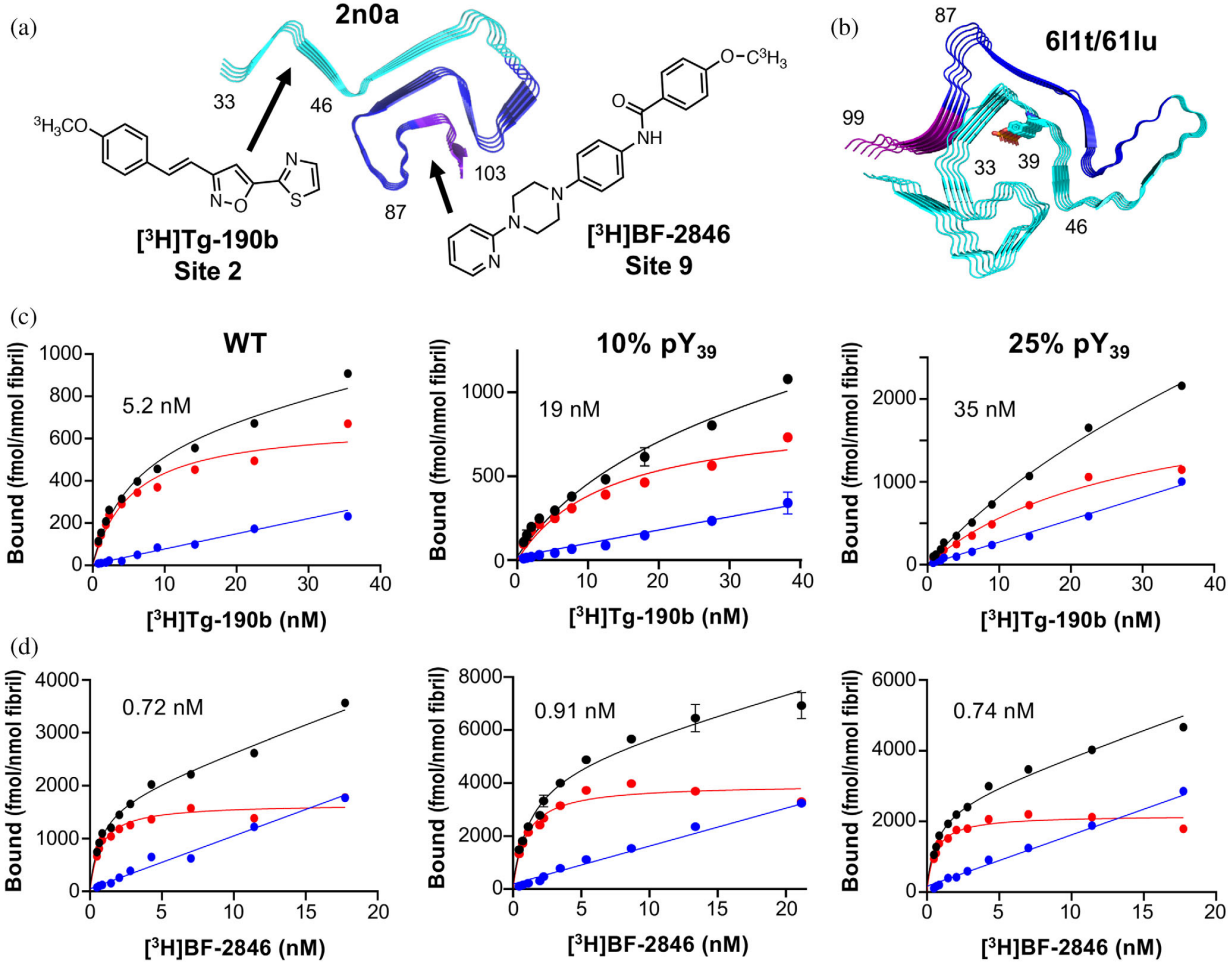
Radioligand binding to fibrils containing Tyr39 phosphorylation (pY_39_) at physiological levels. Site 2 (residues 33–46) and Site 9 (residues 87–103) shown on the (a) solid state NMR structure of wild‐type (WT) alpha‐synuclein (αS) fibrils (PDB ID: 2n0a) and (b) the cryo‐electron microscopy structure of 100% αS‐pY_39_ fibrils (PDB IDs: 6l1t/6l1u) with the structures of Site 2 selective ligand Tg‐190b and Site 9 selective ligand BF‐2846. Saturation binding of [^3^H]Tg‐190b (c) and [^3^H]BF‐2846 (d) radioligands to fibrils made with 100% WT αS, 10% αS‐pY_39_, or 25% αS‐pY_39_ shows that Site 2 is perturbed while Site 9 is not.

Fibrils were prepared from 100% WT αS or mixtures with 10% or 25% αS‐pY_39_. Binding of Tg‐190b, the Site 2 binder, was significantly disturbed by the presence of pY_39_ with the apparent dissociation constant (*K*_d_) increasing from 5.2 to 19 to 35 nM with increased phosphorylation (Figure 5), whereas the *K*_d_ BF‐2846, the Site 9 binder, was about 1 nM for all three fibril samples. This suggests that at physiological stoichiometries, αS‐pY_39_ fibrils have a similar structure to that of WT fibrils in the Site 9 region, with only local effects around Site 2. This finding is incompatible with the broad conformational rearrangement observed by Zhao et al. in 6l1t/6l1u or mixtures of the 2n0a and 6l1t/6l1u fibril polymorphs, both of which would dramatically change Site 9 binding since the pocket around Phe94 is eliminated in 6l1t/6l1u (Figure 5). Indeed, the 10‐fold change in *K*_d_ for Site 2 also seems to be less dramatic than what one would expect for Tg‐190b binding to the 6l1t/6l1u polymorph, since pY_39_ is buried in the fibril core, stabilized by salt bridge interactions with several Lys residues. While further investigations using solid state NMR or cryo‐EM will be necessary to fully understand the structural basis for these effects on ligand binding, these experiments demonstrate the value of accessing modified αS to investigate the impact of physiological PTMs on ligand binding.

### 3.2 Biophysical characterization of vesicle binding of αS‐pS_87_

Phosphorylation at Ser87 has been studied by multiple groups, with a focus on aggregation–the Lashuel group showed that pS_87_ levels are increased in synucleinopathy patient brains and performed in vitro and rat studies, where it slowed down and reduced aggregation, resulting in less toxicity (Paleologou et al., 2010). Notably, they used either mutational mimics or enzymatic phosphorylation by kinase CK1 coupled to a S_129_A mutation to block phosphorylation at that site. The Churchill Group used GCE and incorporated authentic pS_87_ modification with minimal sequence scars from TEV cleavage, which exhibited dopamine or Cu^2+^‐induced oligomerization trends that were similar to WT αS but significantly increased toxicity in the SH‐SY5Y neuronal cell model (Ha et al., 2014). Lastly, the Li and Liu groups performed protein semi‐synthesis, resulting in a completely authentic αS‐pS_87_ construct, and their cryo‐EM study showed its unique fibril structure—unlike the case of pY_39_, pS_87_ could not be stabilized by nearby positively charged residues and causes the broader C‐terminal region of αS to be excluded from the fibril core while including the entire N‐terminal region (PDB: 8jey) (Hu et al., 2024).

On the other hand, the effects of pS_87_ on the native role of αS are relatively understudied: the Lashuel group studied αS conformation in the presence and absence of lipid membranes using circular dichroism, indicating that pS_87_ alters the conformation of the helix that forms when bound to sodium dodecyl sulfate micelles and that it reduces helicity on vesicles (Paleologou et al., 2010). Again, they used the proteins modified with mutational mimics/blocks and enzymatic modification for this.

**FIGURE 6:**
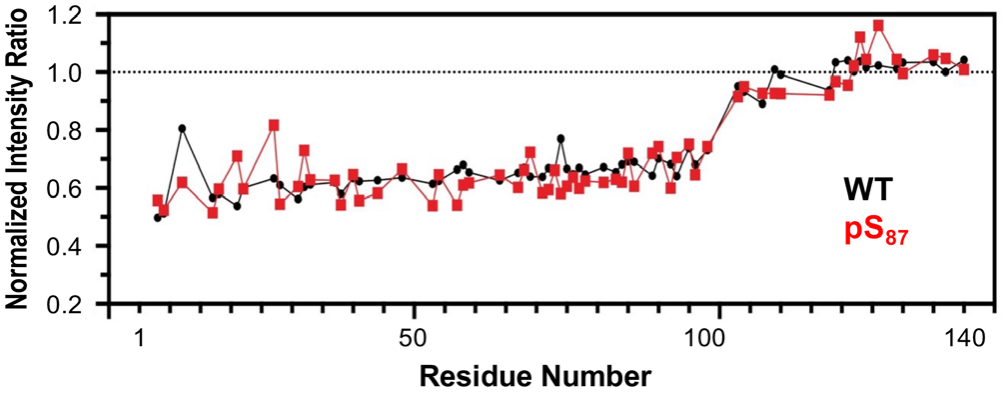
Effects of Ser87 phosphorylation (pS_87_) on vesicle binding, investigated by NMR. NMR intensity ratios for each residue were calculated from proton‐nitrogen heteronuclear single quantum coherencespectra collected with ^15^N‐labeled alpha‐synuclein variants in the presence or absence of small, unilamellar vesicles, normalized by the average ratio for residues 101–140. No significant differences in binding are seen. WT, wild‐type.

To understand the effects of authentic pS_87_ on vesicle binding, we first acquired proton‐nitrogen heteronuclear single quantum coherence spectra (^1^H,^15^N‐HSQC) in the presence and absence of small, unilamellar vesicles (SUVs) that are composed of 60:25:15 1,2‐dioleoyl‐sn‐glycero‐3‐phosphocholine/1,2‐dioleoyl‐sn‐glycero‐3‐phosphatidylethanolamine/1,2‐dioleoyl‐sn‐glycero‐3‐phospho‐L‐serine (DOPC/DOPE/DOPS). The NMR resonance chemical shift changes between spectra for free WT αS or αS‐pS_87_ were very minimal (Figure S9). We observed that both pS_87_ and WT αS led to a ~60% reduction of intensity for resonances corresponding to αS residues ~1 to 100 (Figure 6). This reduction is caused by the binding of this region to vesicles, which are slowly tumbling. The lack of any difference in the binding profiles suggests that pS_87_ does not substantially affect vesicle binding under these conditions.

**FIGURE 7:**
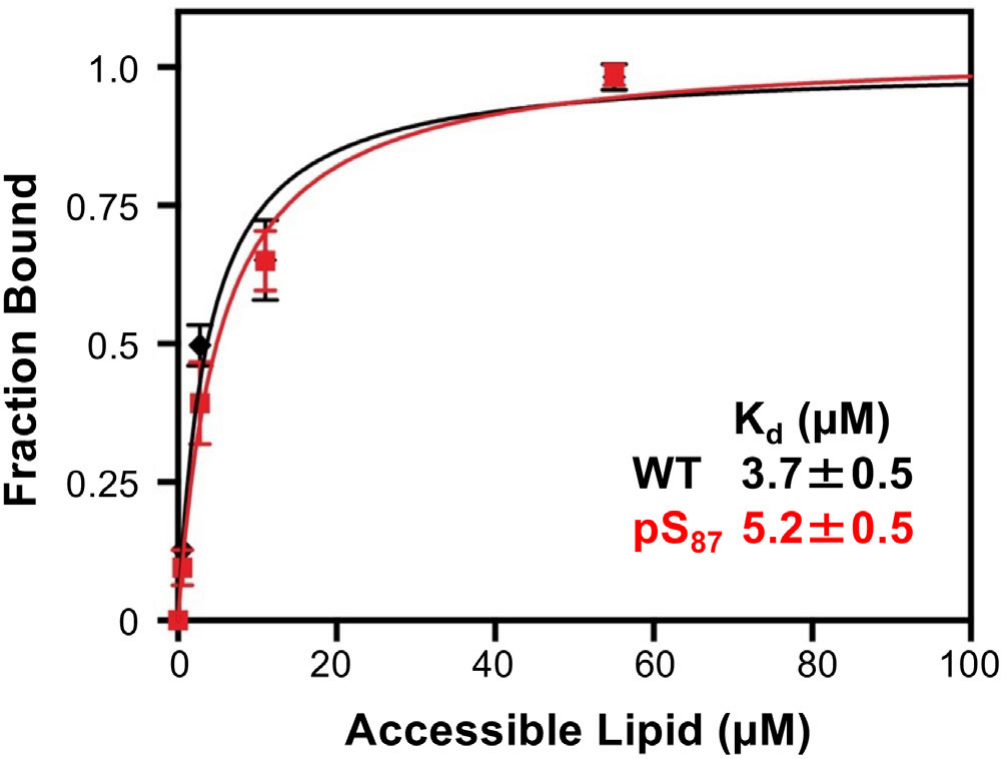
Effects of Ser87 phosphorylation (pS_87_) on vesicle binding, investigated by fluorescence correlation spectroscopy. Alpha‐synuclein constructs labeled with Atto488 were examined in the presence of varying concentrations (1–50 μM accessible lipid) of lipid vesicles consisting of 50:50 1‐palmitoyl‐2‐oleoyl‐sn‐glycero‐3‐phospho‐L‐serine/1‐palmitoyl‐2‐oleoyl‐glycero‐3‐phosphocholine, showing no significant effect on the apparent *K*_d_. WT, wild‐type.

To rigorously determine the effect of pS_87_ on the αS‐vesicle *K*_d_, we performed FCS, which is a well‐established method for quantifying biomolecule interactions, including αS‐vesicle binding (Rhoades et al., 2006). FCS and NMR are complementary techniques in this context: FCS provides quantitative measurements with a slight perturbation to the protein, while NMR offers qualitative insights and low‐resolution structural information without perturbation. It is also notable that this binding affinity measurement could not be completed when we previously generated pS_87_ by chemical protein synthesis due to low yields (Galesic et al., 2023). For this experiment, we used fluorescently labeled αS variants with a Cys mutation at site 114, αS‐C^488^_114_ and αS‐pS_87_C^488^_114_ (denoted WT αS and pS87, respectively, in Figure 7). We prepared lipid vesicles composed of 50:50 1‐palmitoyl‐2‐oleoyl‐sn‐glycero‐3‐phospho‐L‐serine/1‐palmitoyl‐2‐oleoyl‐glycero‐3‐phosphocholine (POPS/POPC). The diffusion times of the free αS proteins and vesicles were initially measured. To evaluate αS‐vesicle binding, a fixed amount of labeled αS construct was added to varying concentrations of vesicles, and the fraction of protein bound was determined by fitting a two‐component autocorrelation function. The fraction bound at each vesicle concentration was then used to generate a binding curve for each αS construct. We observed no significant difference in vesicle binding affinity caused by phosphorylation at Ser87 (Figure 7; *K*_d,app_(WT αS) = 3.7 ± 0.5 μM, *K*_d,app_(pS_87_) = 5.2 ± 0.5 μM). This result is consistent with our observations in the NMR vesicle binding experiments, showing the complementarity of these methods, both using PTM‐modified αS constructs that are difficult to access through NCL.

### 3.3 Tau acetylation effects on microtubule polymerization

**FIGURE 8:**
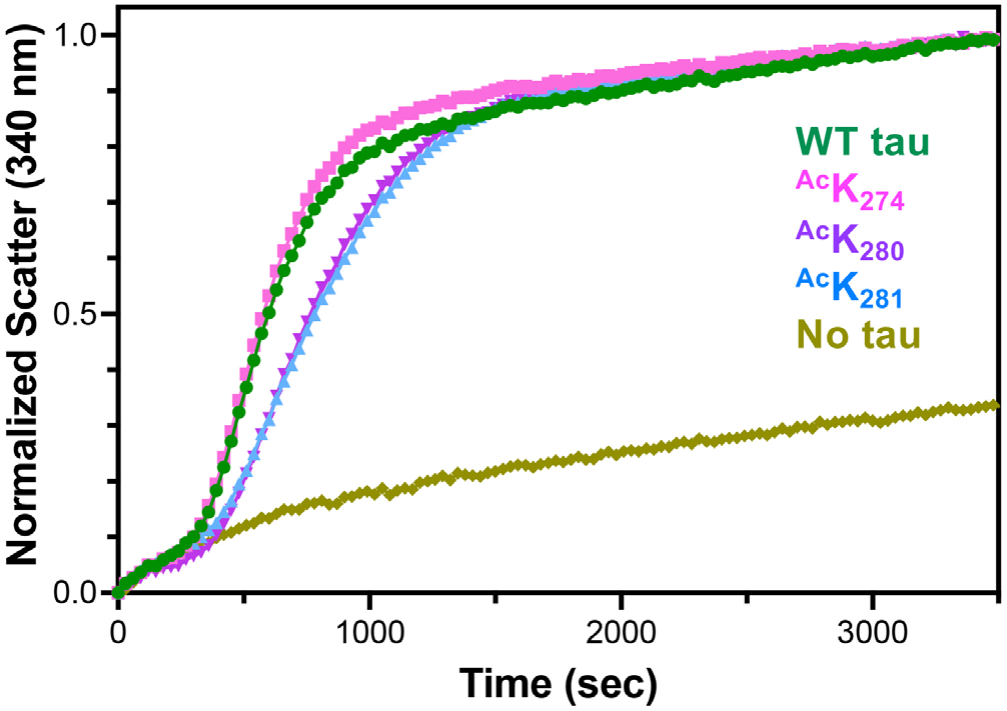
Microtubule polymerization assay with acetylated tau. Wild‐type (WT) 0N4R tau (green) showed comparable polymerization rates to tau‐^Ac^K_274_ (pink). Tau‐^Ac^K_280_ (teal), and tau‐^Ac^K_281_ (blue) both showed slightly impaired polymerization compared to WT tau. Tubulin/GTP‐only (mustard) was included as a negative control experiment. Polymerization data are an average of three replicates per day with three overall replicates.

K_274_, K_280_, and K_281_ have been identified as pathologically relevant sites through MS studies of diseased tissue. It is critical to investigate the potential functional and regulatory roles of acetylation in tau‐microtubule interactions. Thus, microtubule polymerization assays were conducted on the three acetylated 0N4R tau constructs generated using GCE (^Ac^K_274_, ^Ac^K_280_, and ^Ac^K_281_), as well as unmodified 0N4R tau (WT tau). These polymerizations were conducted at 37°C, a constant guanosine triphosphate (GTP) concentration, and a 1:2 concentration ratio of tau to tubulin. Light scatter at 340 nm was used to quantify the increasing turbidity of the solution as microtubule polymerization occurred. Spontaneous tubulin/GTP polymerization in the absence of tau was measured to serve as a control. The ^Ac^K_274_ variant induced polymerization at a similar rate to WT tau, but both ^Ac^K_280_ and ^Ac^K_281_ showed a slight reduction in the polymerization rate (Figure 8).

Although each construct had only a single acetylation site, we were surprised to observe a greater impairment in polymerization rate for ^Ac^K_280_/^Ac^K_281_ compared to ^Ac^K_274_. These results suggest a connection to the resolved near‐atomic cryo‐EM model of the microtubule‐tau complex by Kellogg et al. which demonstrated that K_281_ plays a crucial role in the complex (Kellogg et al., 2018). Further structural work by Brotzakis et al. quantified tau‐microtubule contact interactions and found that K_274_ has many fewer contacts than K_280_ and K_281_, suggesting that acetylation of those two sites would have a larger impact on the microtubule‐tau complex, as observed in the polymerization kinetics (Brotzakis et al., 2021). Additionally, Yan et al. (2022) demonstrated that in HEK293T cells co‐transfected with tau K_280_Q or K_281_Q and USP11, there was reduced polyubiquitin, whereas K_274_Q cells showed no change. This suggests that the ^Ac^K_280_ and ^Ac^K_281_ sites might have a more pathological role, while ^Ac^K_274_ may act as a regulatory buffer depending on the need for acetylation or ubiquitination. Yan et al. also found that USP11 siRNA fully prevented the increase in ^Ac^K_281_ and partially suppressed the increase in ^Ac^K_274_ induced by inhibition of lysine deacetylase SIRT1, highlighting the distinct roles of the 280/281 sites versus the 274 site (Yan et al., 2022).

### 3.4 Effect of pS129 on size distribution and dynamics of αS in αS/tau co‐condensates in vitro

**FIGURE 9:**
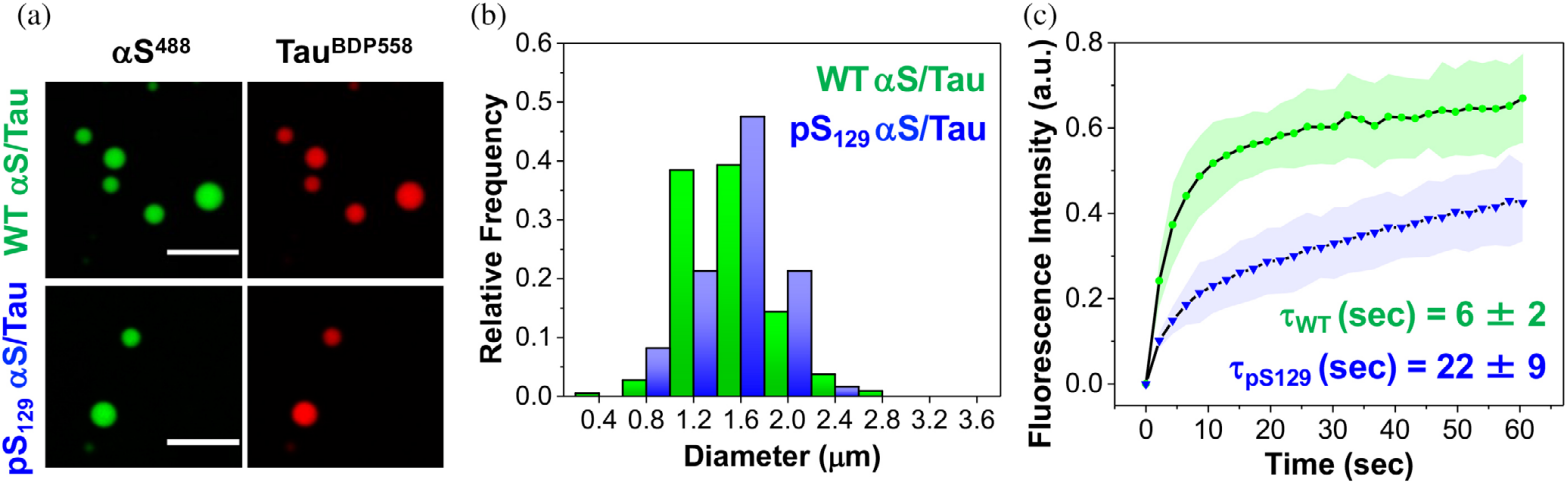
Effect of Ser129 phosphorylation (pS_129_) on size distribution and dynamics of alpha‐synuclein (αS) in αS/tau co‐condensates. (a) Representative images of WT αS/tau and Ser129 phosphorylation (pS_129_) αS/tau (40 μM αS/20 μM tau) condensates in liquid/liquid phase separation (LLPS) buffer, doped with 5% αS‐C^488^_9_ or 5% αS‐C^488^_9_pS_129_, and 5% tau^BDP558^. (b) Size distribution of wild‐type (WT) αS/tau and pS_129_ αS/tau after 1 h of incubation. Condensate diameters were measured using Image J. (c) Fluorescence recovery after photobleaching profile showing the translational mobility of WT αS or pS_129_ αS in αS/tau co‐condensates (doped with 5% αS‐C^488^_9_ or 5% αS‐C^488^_9_pS_129_). Error bars represent the standard deviation of three measurements. Ex: 488 nm and Em: 500–540 nm for Alexafluor 488‐labeled αS, Ex: 561 nm and Em: 570–620 nm for tau^BDP558^. Scale bars are 5 μm.

As a final example of biophysical studies enabled by the production of PTM‐modified proteins, we investigated αS/tau co‐condensate formation. Recently, αS has been shown to interact with tau through electrostatic attractions, forming LLPS condensates between the highly negatively charged C‐terminal region of αS and the positively charged proline‐rich region of tau (Gracia et al., 2022). To examine the impact of the pS_129_ modification on the size distribution and dynamics of αS in co‐condensates with tau, we incubated αS‐C^488^_9_ (WT αS) or αS‐C^488^_9_pS_129_ (pS_129_ αS) with BODIPY 558‐labeled 0N4R tau (tau^BDP558^), with each labeled protein present in a 5:95 mixture with the corresponding unlabeled construct (Figure 9). Using two‐color confocal microscopy, we confirmed the formation of αS/tau co‐condensates, detecting αS in the 500–540 nm channel and tau in the 570–620 nm channel. The size distribution of WT αS/tau and pS_129_ αS/tau co‐condensates showed no significant difference, with average diameters of 1.33 ± 0.36 and 1.36 ± 0.34 μm, respectively. However, fluorescence recovery after photobleaching (FRAP) revealed that pS_129_ modification alters the dynamics of αS within αS/tau co‐condensates. Specifically, pS_129_ αS exhibited a significantly slower fluorescence recovery time (*τ*_FR_ = 22.32 ± 8.73 s) compared to WT αS (*τ*_FR_ = 6.03 ± 1.67 s).

## 4 CONCLUSION

In this work, we have described the production and characterization of αS and tau with authentic PTMs introduced by GCE or enzymatic modification. These methods allow for site‐specific incorporation of phosphorylation and acetylation, while being broadly accessible to biochemistry laboratories that may lack peptide synthesis expertise. Additionally, we demonstrated the utility of these approaches for isotopic and fluorescent labeling, allowing for structural and biophysical studies. While Phos‐tag gel electrophoresis provided a useful tool for assessing phosphorylation status, we also highlighted the need for careful interpretation, since band shifts can be influenced by the intrinsic charge of the phosphorylated region as observed for αS‐pS_129_. Beyond protein production, we examined the biophysical and functional consequences of these modifications, particularly focusing on phosphorylation at Ser87, Ser129, and Tyr39 in αS and acetylation at Lys274, Lys280, or Lys281 in tau.

By integrating radioligand binding studies with PTM‐modified fibrils, we demonstrated how PET probe interactions can serve as a functional readout of structural changes induced by modifications. Our findings suggest that pY_39_, when present at physiological sub‐stoichiometric ratios, perturbs PET probe binding only at Site 2, likely reflecting blocked interactions by phosphorylation and localized, but not global, structural changes of the fibrils. Future work involving detailed structural characterization will be essential for understanding the underlying molecular basis of this altered binding affinity. Additionally, since pY_39_ interacts with K_21_ and K_34_ in the 6l1t/6l1u structure, investigating the impact of Lys acetylation at these sites on fibril conformation and radioligand binding will also be of interest, particularly if both PTMs can be incorporated into the same construct.

We also examined how PTMs influence αS's native functions, including membrane interactions. Vesicle binding studies using NMR and FCS showed that pS_87_ does not significantly impact αS‐vesicle interactions. This finding contrasts with previous studies using phosphomimetic mutations or enzyme‐blocking mutations, which showed that this PTM reduces helicity of the protein on vesicles (Paleologou et al., 2010). This highlights the importance of employing authentic modifications rather than relying on mimics when studying PTM effects. As noted above, investigation of Lys acetylation in αS is also of interest, and this charge‐neutralizing PTM would be expected to inhibit αS interactions with vesicles, at least for some sites.

In the case of tau acetylation, we observed a greater impairment in polymerization rate for ^Ac^K_280_/^Ac^K_281_ compared to ^Ac^K_274_, consistent with literature reports suggesting that the ^Ac^K_280_ and ^Ac^K_281_ sites might have a more pathological role. One interpretation of acetylation effects is that they prevent ubiquitination and thus protein degradation while also impairing interaction with microtubules, making tau more accessible for phosphorylation and subsequent aggregation. Indeed, Yan et al. found that inhibition of the deacetylase HDAC6 by tubastatin A increased tau KXGS motif phosphorylation at S262 (Yan et al., 2022). Acetylation at KXGS tau motifs may inhibit phosphorylation, suggesting a protective role of one PTM over another, potentially leading to aggregation (Keller et al., 2000; Reynolds et al., 2000). Investigation of such cross‐talk between PTMs is another exciting opportunity afforded by our GCE methods, and efforts are underway to apply the pSer‐3.1G system to tau phosphorylation.

Finally, we demonstrated that pS_129_ modification does not affect the size of αS/tau co‐condensates but significantly reduces αS mobility, showing a role in modulating condensate dynamics. Additionally, the impact of pS_129_ modification on αS dynamics provides a foundation for understanding how phosphorylation influences transitions from condensates to mature fibrils, highlighting a role in the underlying mechanisms of amyloid aggregation.

Overall, this work showcases the value, as well as some challenges, of GCE and enzymatic methods for producing PTM‐modified proteins and their application in biophysical and structural characterization. With these tools, we provide new insights into the role of acetylation and phosphorylation in αS and tau, contributing to a deeper understanding of PTM‐mediated regulation in neurodegenerative disease pathology. There are also clear opportunities to use GCE and enzymatic modification together to achieve incorporation of two or more PTMs in the same protein, which will allow for exploration of the most impactful and physiologically relevant combinations of PTMs. These authentic modifications can also be compared to phosphomimic (Asp and Glu) and acetylmimetic (Gln) variants. Finally, while we have focused on avoiding methods like NCL to ensure that these techniques are as accessible as possible, using GCE and enzymatic modifications in combination with chemical peptide synthesis and NCL can allow one to efficiently access very highly modified proteins, as we have shown in some contexts with αS and look forward to systematic applications in both αS and tau (Haney et al., 2016; Tanaka et al., 2013).

## AUTHOR CONTRIBUTIONS

**Ibrahim G. Saleh:** Investigation; writing – original draft; writing – review and editing. **Marie Shimogawa:** Conceptualization; investigation; writing – original draft. **Jennifer Ramirez:** Conceptualization; investigation; writing – original draft. **Bernard Abakah:** Investigation. **Yarra Venkatesh:** Investigation; writing – review and editing; resources. **Honey Priya James:** Investigation; resources. **Ming‐Hao Li:** Investigation; resources. **Sarah A. Louie:** Investigation. **Marshall G. Lougee:** Investigation. **Wai‐Kit Chia:** Investigation; resources. **Christopher Brue:** Investigation; resources. **Richard B. Cooley:** Supervision; writing – review and editing. **Ryan A. Mehl:** Supervision; funding acquisition; writing – review and editing. **Tobias Baumgart:** Funding acquisition; supervision; writing – review and editing. **Robert H. Mach:** Funding acquisition; supervision; writing – review and editing. **David Eliezer:** Funding acquisition; supervision; writing – review and editing. **Elizabeth Rhoades:** Conceptualization; supervision; funding acquisition; writing – review and editing. **E. James Petersson:** Conceptualization; supervision; funding acquisition; writing – original draft; writing – review and editing.

## Supporting information


**Data S1.** Supporting Information.

## Data Availability

The data that support the findings of this study are available from the corresponding author upon reasonable request.
